# Determination of the Effect of Co-cultivation on the Production and Root Exudation of Flavonoids in Four Legume Species Using LC–MS/MS Analysis

**DOI:** 10.1021/acs.jafc.1c02821

**Published:** 2021-08-04

**Authors:** Federico Leoni, Hossein Hazrati, Inge S. Fomsgaard, Anna-Camilla Moonen, Per Kudsk

**Affiliations:** † Group of Agroecology, Institute of Life Sciences, Scuola Superiore Sant’Anna, Piazza Martiri della Libertà, 33, 56127 Pisa, Italy; ‡ Department of Agroecology, Aarhus University, Forsøgsvej, DK-4200 Slagelse, Denmark

**Keywords:** wheat, intercropping, living mulch, root exudate, plant interactions, medicarpin, allelopathy

## Abstract

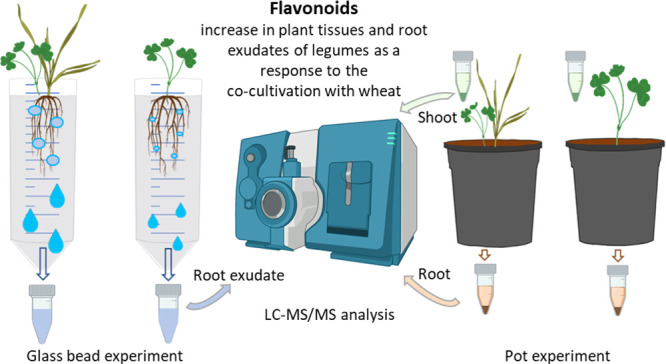

Flavonoids play a key role in the regulation of plant–plant and plant–microbe interactions, and factors determining their release have been investigated in most of the common forage legumes. However, little is known about the response of flavonoid production and release to co-cultivation with other crop species. This study investigated alterations in the concentration of flavonoids in plant tissues and root exudates in four legumes [alfalfa (*Medicago sativa L.*), black medic (*Medicago polymorpha L.*), crimson clover (*Trifolium incarnatum L.*), and subterranean clover (*Trifolium subterraneum L.*)] co-cultivated with durum wheat [*Triticum turgidum* subsp. *durum* (Desf.) Husn.]. For this purpose, we carried out two experiments in a greenhouse, one with glass beads as growth media for root exudate extraction and one with soil as growth media for flavonoid detection in shoot and root biomass, using LC–MS/MS analysis. This study revealed that interspecific competition with wheat negatively affected legume growth and led to a significant reduction in shoot and root biomass compared with the same legume species grown in monoculture. In contrast, the concentration of flavonoids significantly increased both in legume biomass and in root exudates. Changes in flavonoid concentration involved daidzein, genistein, medicarpin, and formononetin, which have been found to be involved in legume nodulation and regulation of plant–plant interaction. We hypothesize that legumes responded to the co-cultivation with wheat by promoting nodulation and increasing exudation of allelopathic compounds, respectively, to compensate for the lack of nutrients caused by the presence of wheat in the cultivation system and to reduce the competitiveness of neighboring plants. Future studies should elucidate the bioactivity of flavonoid compounds in cereal-legume co-cultivation systems and their specific role in the nodulation process and inter-specific plant interactions such as potential effects on weeds.

## Introduction

The need for more sustainable cropping systems has boosted the interest in cropping practices that reduce the reliance on external inputs while maintaining an adequate level of crop productivity.^1^ An intercropping of subsidiary legumes with cereals (living mulch) has been proposed as a promising management strategy to support ecological mechanisms for weed control, pathogen, and pest reduction and optimize nutrient use and soil fertility.^2^

Ecosystem services provided by legumes in living mulch systems can be partially assigned to the biosynthesis and release of secondary metabolites such as flavonoids into the rhizosphere.^3^ The impact of legume-cereal intercropping on flavonoid changes in legume tissues and root exudates remains not well studied, although elucidating how legumes respond to the co-cultivation with wheat can be of high importance to provide possible mechanistic explanation of the multiple beneficial effects reported at the cropping system level of legume-cereal intercropping.^4^

The chemicals released into the soil by roots are commonly defined as root exudates. Through exudation of a wide range of compounds, the roots regulate the soil microbial community in the rhizosphere, encourage beneficial and mutualist symbioses, modify the chemical and physical properties of the soil, and inhibit the growth of competing plant species.^5^ In particular, flavonoids identified in legume root exudates play a crucial role in regulating inter-plant and plant–microbe interactions,^6,7^ and they are pivotal for the activation of nod genes in N-fixing bacteria (node inducer compounds), allowing bacteria to enter plant roots and begin the formation of nodules.^8,9^ Moreover, flavonoids released in the rhizosphere can modify the chemical soil composition and nutrient availability through their activity as reducers or metal chelators. Flavonoids such as kaempferol, nicotiflorin, and rutin have been identified as powerful chelators, and their properties play a key role in nutrient availability, in particular for phosphorus (P) and microelements such as iron (Fe) and manganese (Mn), which are often limited especially under organic farming systems.^6^

Allelopathy has important beneficial implications for agriculture, as in the case of natural weed control. Flavonoids are involved in allelopathic interactions with neighboring plants, and for this purpose, legume subsidiary crops are frequently used as cover crops or in intercropping systems to reduce weed germination and suppress weed growth.^10,11^ The morphological characteristics of legumes do not fully explain their weed suppression ability, and an increasing number of studies have shown that allelopathy also plays a key role.^12,13^ Moreover, recent studies on the root uptake of organic compounds have shown that allelochemicals exuded into the rhizosphere by plant roots could be adsorbed by neighboring plants and translocated into the shoot.^12^ This aspect might be particularly interesting for intercropping systems where plant diversity is high because it offers an interesting perspective for a natural enhancement of plant defense against pathogens.

Biosynthesis of flavonoids in plant tissues is highly regulated by biotic and abiotic factors,^6^ and how flavonoid composition changes in legume biomass and root exudates when legumes are intercropped with non-legume species is not well studied.

The objective of this work was to investigate the concentration and diversity of flavonoids in the biomass and root exudates of four legume species and investigate how the flavonoid profiles changed in response to the co-cultivation with durum wheat.

We hypothesized that the concentration of flavonoids increases in legume shoot and root and root exudates in response to co-cultivation with wheat. In addition, we hypothesized that the flavonoid content of legumes is species specific and that the characterization of legumes based on the flavonoid content can be an additional parameter to consider for the evaluation of suitable legumes for living mulch systems. To test our hypothesis, we carried out two experiments to investigate alteration in the flavonoid composition in the shoot, root, and root exudates in response to co-cultivation with durum wheat. Chemical profiling of root exudates can be challenging due to the lack of suitable and universal techniques for quantifying metabolites present in the root exudate of plants in field and pot experiments.^12^ For this reason, in our study, a soil-free growth system for root exudates extraction was used to prevent the degradation of the compounds by microbes in one of the experiments.

## Methodology

### Chemicals

Kaempferol (96%) and formononetin (99%) were purchased from Fluka(Brøndby, Denmark). Biochanin A (97%), genistin (95%), nicotiflorin (98%), and astragalin (99%) were purchased from Sigma-Aldrich (Brøndby, Denmark). Hyperoside (99%) was purchased from Roth (Karlsruhe, Germany). Daidzein (97%) and genistein (97%) were purchased from Lancaster (Brønshøj, Denmark). Rutin (99%), apigenin (99%), naringenin (99%), and daidzin (90%) were purchased from Extrasynthese (Genay, France). Medicarpin was obtained from Dr. Paul M. Dewick at the University of Nottingham, UK. Quercetin-rha-xyl-gal was isolated from white clover, purified, and identified by its UV, mass, and nuclear magnetic resonance spectra as part of a previous study.^14^

Stock flavonoid solutions of 1 g·l^–1^ were prepared by dissolution in methanol. Working standard solutions of the compounds were obtained by serial dilution of the stock standard solutions in 35% MeOH and 65% Milli-Q water (v/v) containing 0.2% formic acid. Mixed standard curves for the negative and positive modes were generated from 10 concentrations of each standard and used for quantification.

### Glass Bead Experiment

#### Experimental Setup

The method developed by Hazrati et al., 2020,^12^ was used for growing plants. Micro-glass beads of the size of soil particles were used as growth media, allowing plants to develop roots with a morphology like the roots of plants growing in soil.

Fifty milliliter plastic tubes were used as containers for growing plants. A 7 mm drainage hole was made in the base of the tubes and covered with a 7 cm^2^ piece of mesh (pore size 30 μm) to avoid any loss of growth media, to stop the roots from growing out of the tubes, and to prevent contamination of the extracted root exudate by small pieces of root tissue.

Tubes were filled with 40 g of micro-glass beads with a size of 250–425 μm. Colored self-adhesive tape was wrapped around the tubes to prevent algal growth and prevent light exposure to the roots. Seeds of legumes and wheat were pre-germinated on water-saturated Whatman filter papers in Petri dishes (one seed in each Petri dish) placed in a growth chamber at 20 °C with a 16 h photoperiod for 72 h. Germinated seedlings were transplanted into the tubes that were kept in a greenhouse under controlled conditions at a 16/8 h photoperiod and a temperature of 20/16 °C (day/night). Plants were watered every second day with 5 mL of half-strength Hoagland solution with a final watering 2 days before sampling.

Four different legume species, including alfalfa (*Medicago sativa L.* cv. Gamma), black medic (*Medicago polymorpha L.* cv. Scimitar), crimson clover (*T. incarnatum L.* cv. Kardinal), and subterranean clover (*T. subterraneum L.* cv. Mintaro), were grown in monoculture and co-cultivated with durum wheat [*Triticum turgidum* subsp. *durum* (Desf.) Husn. cv. Minosse] to mimic the intercropping scenario.

Four legume species of this study were already studied in living mulch systems with cereals and were chosen to represent a diversity of morphological and physiological characteristics.

The experimental setup included two plants of legume growing alone, two wheat plants growing alone, and two legume plants growing together with two plants of wheat. Each treatment was repeated five times. Growing wheat alone was necessary to be able to single out flavonoids produced exclusively by the legumes.

#### Root Exudate Extraction

The method developed by Hazrati et al., 2020,^12^ was used for extracting root exudates. Root exudate extraction was performed on 3 week-old legume plants (BBCH: 23). A solvent containing 70% methanol (v/v) and 0.2 formic acid (v/v) was used. Fifteen milliliters of the extraction solution were injected with a syringe to the top of the growing media, and vacuum pressure was applied to accelerate the flushing of the solution through the micro-glass beads. Flushing of the growth media for collecting root exudates was performed in 30 s to minimize root cell damage by applied organic solvent. Each tube contained 10 mL of water (from previous irrigations), which, together with the extraction solution, added up to a final collected volume of approximately 25 mL for each tube. The collected solution was filtered through a 0.22 μm syringe filter and transferred into glass vials before liquid chromatography (LC)–(mass spectrometry) MS/MS analysis.

### Pot Experiment

#### Experimental Setup

This experiment was carried out in a greenhouse at the Department of Agroecology of Aarhus University. One liter pots filled with sandy loam field soil (2.8% organic matter, 11.5% clay, 28.4% silt, and 57.2% sand) were used as a growth medium. Plants were grown under controlled conditions at a 16/8 h photoperiod and a temperature of 20/16 °C (day/night). Pots were watered regularly with a sub-irrigation system.

Four cereal-legume combinations were investigated. Durum wheat (cv. Minosse) was grown with the same four legume species as in the glass bead experiment. Legumes as the sole crop were used as control. Plants were sown according to a specific spatial arrangement. Four legume plants, which were the target plants in this experiment, were seeded at the center of each pot, and six plants of wheat were seeded with equal distance around legumes. The four legume plants in monoculture were seeded in the center of each pot as control. Treatments were repeated seven times in a complete randomized block design.

Legumes and wheat were seeded on 10 November 2019. Four week-old legume plants (BBCH:23–25) were harvested, and the roots were washed with distilled water, then carefully separated from the shoots, and immediately immersed in liquid nitrogen to prevent any enzymatic reaction before being transferred to a freezer at −80 °C. Finally, samples were freeze-dried and kept at room temperature until analysis.

#### Metabolite Extraction for Root and Shoot Tissue

Ground root and shoot tissue (20 mg) were transferred into a 2 mL Eppendorf tube (with safe lock), and 1 mL of 80% methanol containing 0.2% formic acid was added instantly. The samples were sonicated with ultrasonication for 45 min and centrifuged at 4500 g (Sigma 1–14 K microcentrifuge, Buch & Holm, Herlev, Denmark) for 10 min.

The addition of solvent, sonication, and centrifugation was repeated. The samples and the insoluble plant materials were discarded, and the supernatant was transferred to dark 4 ml glass vials. The (500 μL) supernatant was sampled and diluted 2 and 100 times in 100% Milli-Q water to obtain concentrations within the calibration curve points. The diluted mixture was filtered through a 0.22 μm cellulose acetate syringe filter before the LC–MS/MS analysis.

### Quantification of Flavonoids in Plant Material and Root Exudate by LC–MS/MS

Identification and quantification of flavonoids were performed using LC–MS/MS methods developed by Hazrati et al., 2021.^3^ Some of the analytes of interest were only charged either in positive or in negative mode. Therefore, two LC–MS/MS methods were used. A reversed-phase Synergi Fusion-C18, 80A column (250 × 2 mm id, 4 μm particle size) was used for both modes to separate the analytes. The mobile phase for both modes was a binary solvent mixture composed of solvent A (100% Milli-Q water with 0.2% formic acid) and solvent B (100 acetonitrile with 0.2% formic acid) with a flow rate of 0.35 mL min^–1^ and an injection volume of 30 μm. The LC conditions for (ESI -) are as follows: the column oven was set at 40 °C; curtain gas, 30 psi; ion spray voltage, −4200 V; temperature, 560 °C; ion source gas 1, 60 psi; and ion source gas 2, 50 psi. The binary gradient for ESI is as follows: 0–3 min, column equilibration (80% A), 3–24 min, ramping to (45% A), 24-26 min, reduced to (0% A), 26-29 min, isocratic hold (0% A), 29–29.5 min, increased to (80% A), 29.5–40 min, and hold (80% A). Nitrogen gas was used as a collision gas to generate MS/MS fragmentation. The LC conditions for (ESI +) optimized are as follows: the column oven was set at 40 °C; curtain gas, 30 psi; ion spray voltage, −4500 V; temperature, 500 °C; ion source gas 1, 60 psi; and ion source gas 2, 50 psi. The binary gradient for ESI was 0–3 min, column equilibration (80% A), 3–4 min, ramping to (45% A), 4–11 min, reduced to (25% A), 11–12 min, reduced to (0% A) 12–15 min, isocratic hold (0% A), 15–15.5 min, increased to (80% A), 15.5–25 min, and hold (80% A). All other LC–MS/MS parameters were as previously described by Hazrati et al., 2021.^3^ Analyst Software (version 1.6.2) was used for instrument control, data acquisition, and subsequent quantification. Quantification was done on the basis of standard curves prepared in the range of 0.39–400 ng·mL^–1^. Data points of the standard curves were weighted according to ×–1.

### Statistical Analysis

Data analysis was performed using R for statistical computing.^15^ Statistical models were performed using the “Lme4” package for R.^16^ For significant explanatory variables, Tukey’s post-hoc test was performed to separate means (*P* < 0.05) using the “emmeans” package for R.^17^ The normality and homogeneity of residual variance were studied for the validation of each model.

A general linear model (GLM) with Gamma distribution and log link function were used to evaluate the effects of wheat-legume co-cultivation on flavonoid concentrations in root exudates. The model used was
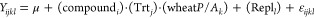
where *Y*_*ijkl*_ is the concentration of flavonoid compounds *i* (compound_*i*_) detected in the root exudate of each legume *j* (Trt_*j*_) cultivated alone or with wheat (in the presence or absence of wheat, wheat*P*/*A*_*k*_). Repl_*l*_ is the replication, μ is the grand mean, and ϵ_*ijkl*_ is the residual error. In the case of significant compound_*i*_·Trt_*j*_·wheat*P*/*A*_*k*_ interaction, the effects of co-cultivation between legume and wheat were investigated separately for each compound and legume species (*P* < 0.05).

For the pot experiment, a GLM with Gamma distribution and log link function were used to evaluate the effects of wheat-legume co-cultivation on flavonoid concentration in legume root and shoot tissue. The model is formulated as
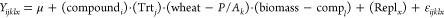
where *Y*_*ijklx*_ is the concentration of flavonoid compound *i* (compound_*i*_) detected in legume species *j* (Trt_*j*_) when they are cultivated alone or with wheat (in the presence or absence of wheat, wheat – *P*/*A*_*k*_) and for each biomass component (root and shoot, biomass – comp_*l*_). Repl_*x*_ is the replication, μ is the grand mean, and ϵ_*ijklx*_ is the residual error. In the case of significant compound_*i*_·Trt_*j*_·*wheat* – *P*/*A*_*k*_·biomass – comp_*l*_ interaction, the effect of co-cultivation between legume and wheat was investigated separately for each compound, legume species, and tissue component (*P* < 0.05).

## Results

### Flavonoid Composition in Legume Root Exudates in Response to Co-cultivation with Wheat

**Figure 1:**
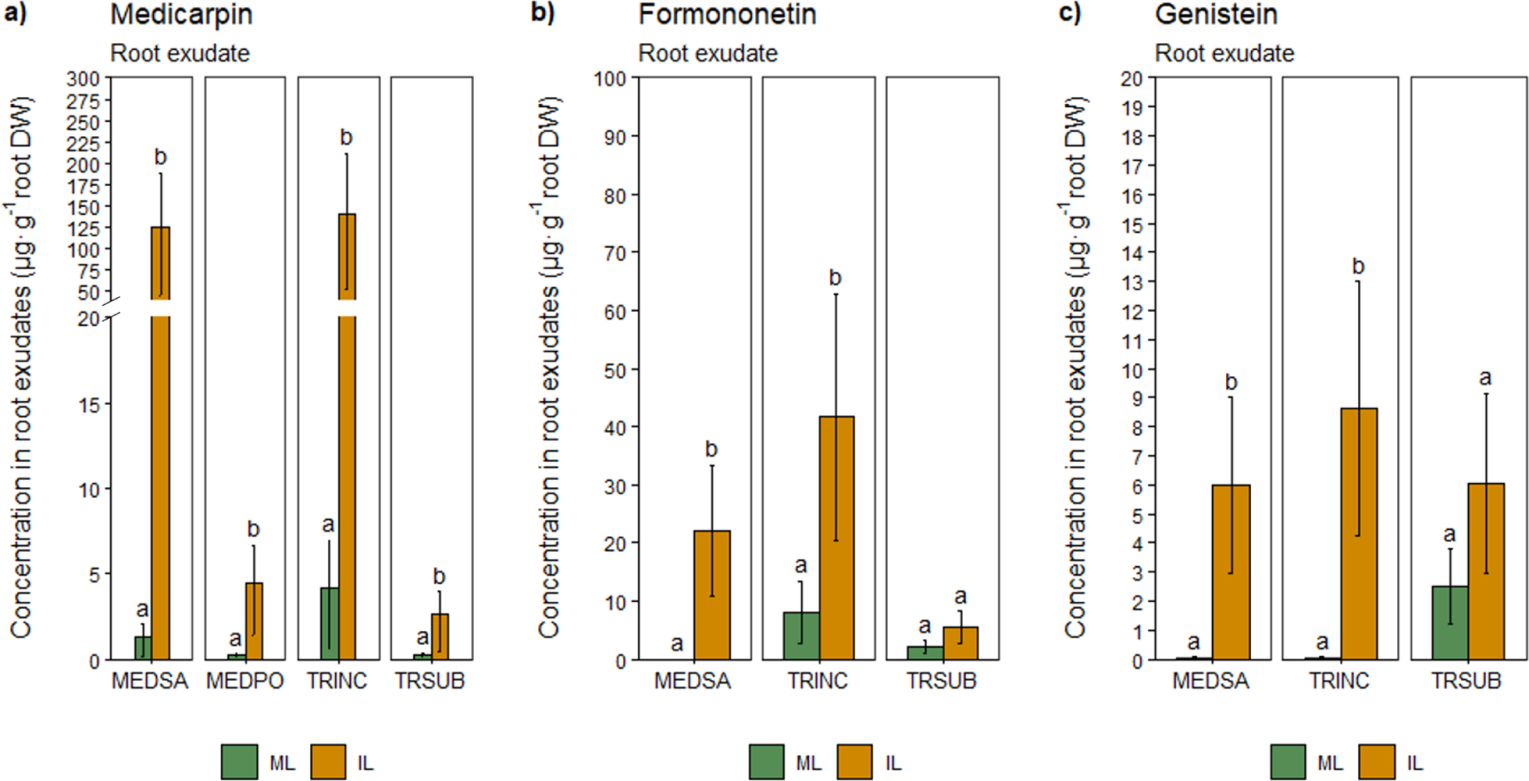
Effects of wheat-legume co-cultivation on (a) medicarpin, (b) formononetin, and (c) genistein in legume root exudates (mean ± SE μg·g^–1^ DW). ML: monocropped legume, IL: intercropped legume. MEDSA: alfalfa, MEDPO: black medic, TRINC: crimson clover, and TRSUB: subterranean clover. Different letters (a,b) for each legume species indicate significant differences at the 0.05 level (Tukey’s post-hoc test). Error bars represent standard error (S.E.).

Medicarpin, formononetin, and genistein were the predominant flavonoids found in legume root exudates. Medicarpin was present in all legume species used in this experiment, whereas formononetin and genistein were detected only in alfalfa, crimson clover, and subterranean clover (Figure 1).

Flavonoid concentrations significantly increased in legume root exudates in response to the co-cultivation with wheat. In particular, the concentration of medicarpin was significantly higher in the exudate of all the co-cropped legume species compared to legumes grown alone. Concentrations of formononetin and genistein significantly increased in alfalfa and crimson clover root exudates due to co-cultivation (Figure 1).

### Legume and Wheat Biomass

**Figure 2:**
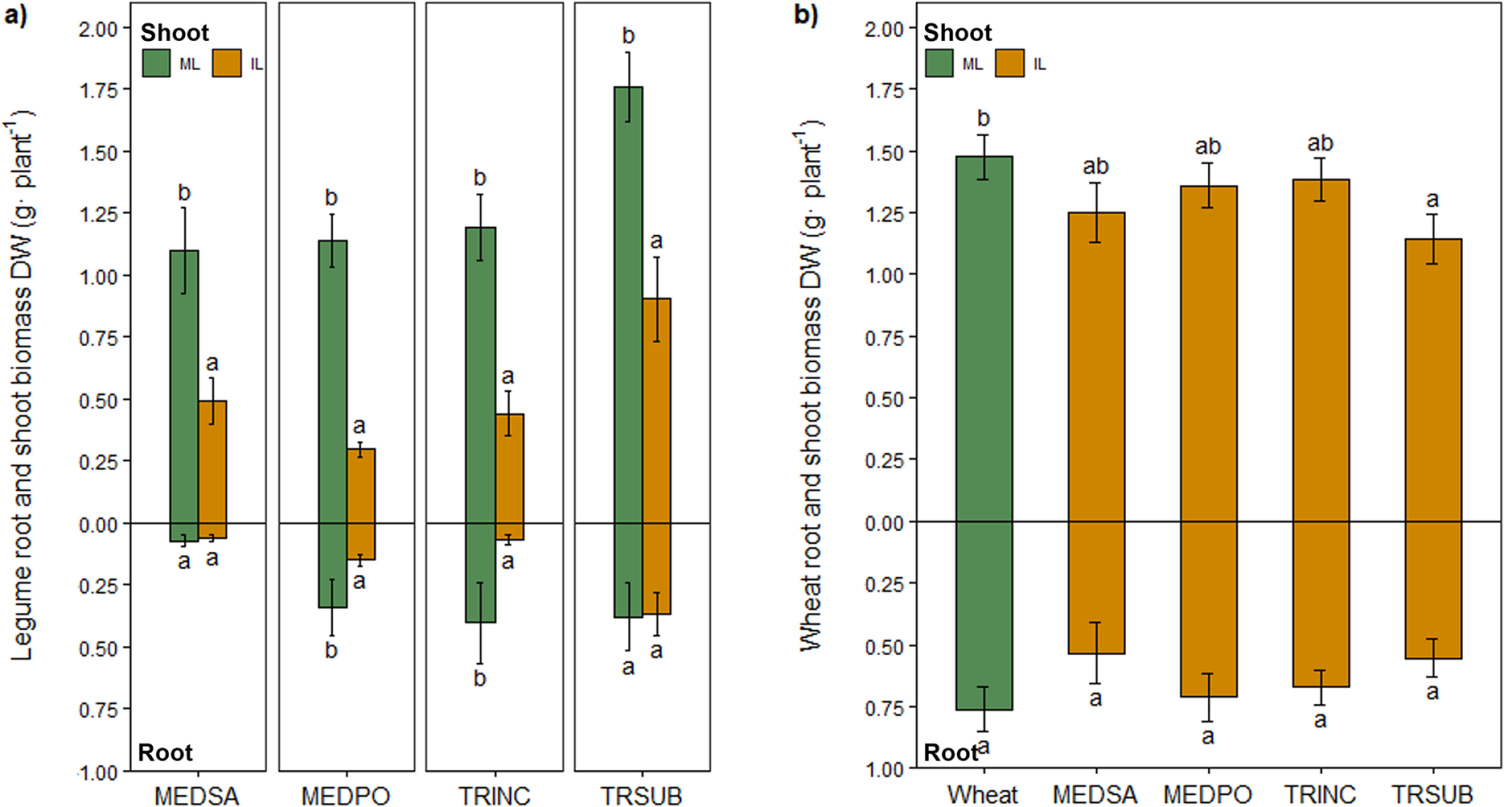
Effects of wheat-legume co-cultivation on root and shoot biomass. ML: monocropped legume, IL: intercropped legume. MEDSA: alfalfa, MEDPO: black medic, TRINC: crimson clover, and TRSUB: subterranean clover. Different letters (a,b) indicate significant differences at the 0.05 level (Tukey’s post-hoc test). Error bars represent S.E.

Co-cultivation of legumes with wheat negatively affected the legume biomass. The decrease in shoot biomass was more pronounced than roots. The shoot biomass of all the intercropped legume species significantly decrease compared to legumes grown in monoculture. A significant reduction in the root biomass of black medic and crimson clover was observed, but no significant changes were detected for alfalfa and subterranean clover (Figure 2a).

In general, co-cultivation with legumes slightly affected wheat biomass except for subterranean clover that significantly reduced the wheat shoot biomass compared to the control (Figure 2b).

### Flavonoid Composition in Legume Root and Shoot Tissue in Response to Co-cultivation with Wheat

Legume species used in this experiment were selected for genetic diversity, which resulted in a distinctly different amount of flavonoids in the plants grown in monoculture. In crimson clover and subterranean clover roots, the concentration of flavonoids was about 15-folds higher than alfalfa and black medic (Table 1). Subterranean clover had the highest concentration of flavonoids in shoot, and it was respectively 6-, 20-, and 21-folds higher than black medic, alfalfa, and crimson clover (Table 1).

**Table 1:** Total Flavonoids in Legume Root and Shoot Biomass in Monocropping and Intercropping Systems and Their Percentage Variation^a^

	total flavonoids (μg·g–1)	total flavonoids (μg·g–1)	
legume	monocropping	intercropping	Δ (%)
Root			
MEDSA	7.53 ± 0.65 a	39.52 ± 3.41 b	+424.83***
MEDPO	6.23 ± 0.53 a	21.19 ± 1.83 a	+240.12***
TRINC	97.26 ± 8.40 b	101.04 ± 8.73 c	+3.88 n.s.
TRSUB	102.50 ± 8.85 b	146.43 ± 12.65 d	+42.85**
Shoot			
MEDSA	84.78 ± 21.83 a	83.48 ± 7.25 a	–1.53 n.s.
MEDPO	251.34 ± 7.36 b	588.04 ± 51.07 c	+133.96***
TRINC	78.55 ± 6.82 a	121.74 ± 10.57 b	+54.98***
TRSUB	1690.15 ± 146.79 c	2388.84 ± 207.48 d	+41.34**

a Different letters (a,b) indicate significant differences at the 0.05 level (Tukey post-hoc test).*** (*P* < 0.001), ** (*P* < 0.01), * (*P* < 0.05), and n.s (*P* > 0.05).

Co-cultivation of legumes with wheat significantly increased the total flavonoid content in legume root and shoot. The concentration of total flavonoids in roots significantly increased in alfalfa, black medic, and subterranean clover by 424, 240, and 42%, respectively, compared to the legume grown alone (Table 1). In legume shoot, the concentration of total flavonoids significantly increased in black medic, crimson clover, and subterranean clover by 133, 54, and 41%, respectively, compared with the same species grown in monoculture (Table 1).

**Figure 3:**
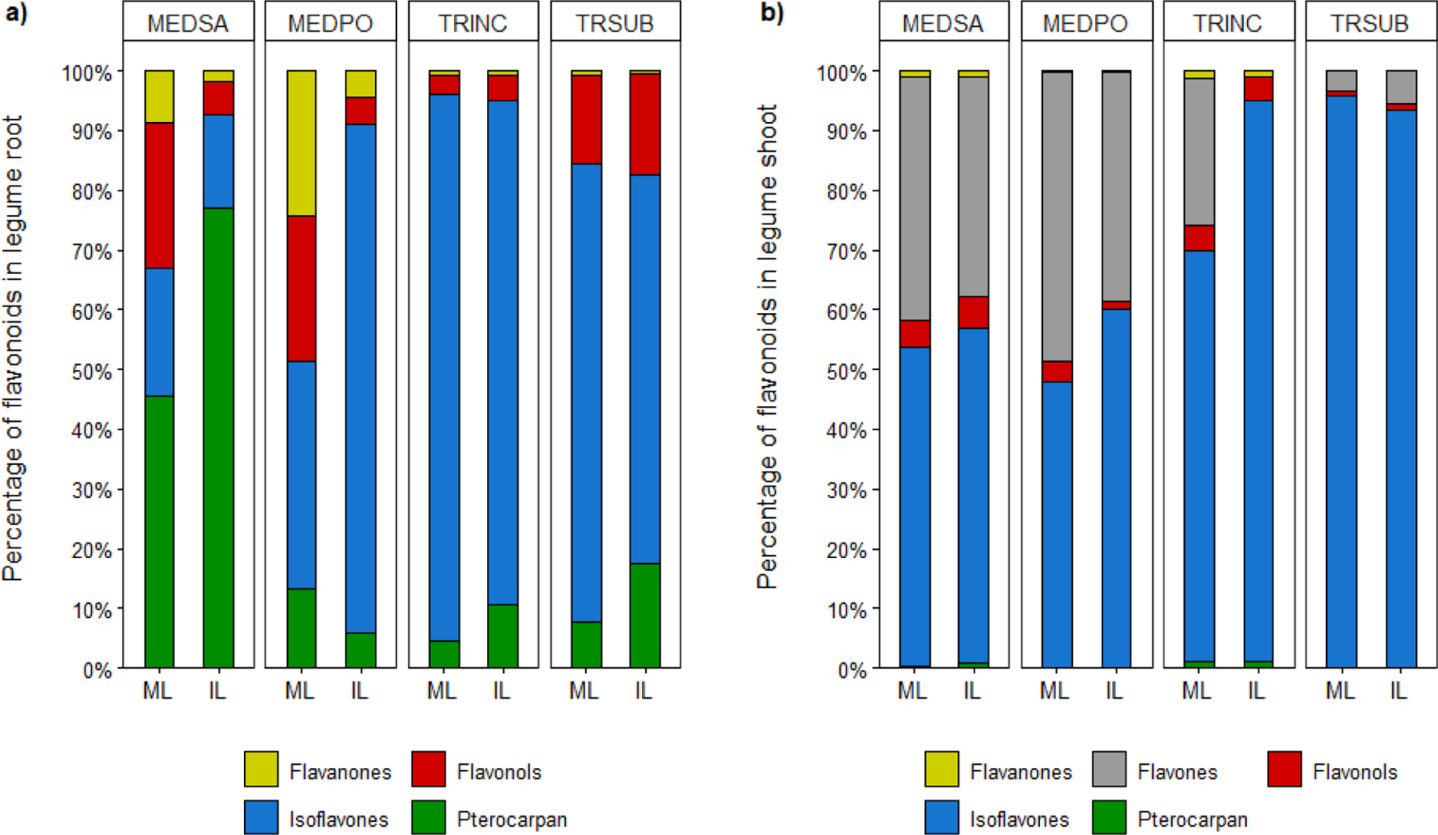
Percentage of flavonoid groups in (a) legume root tissue and (b) legume shoot tissue. ML: monocropped legume, IL: intercropped legume. MEDSA: alfalfa, MEDPO: black medic, TRINC: crimson clover, and TRSUB: subterranean clover.

Flavonoid groups detected in this study included flavanones, flavonols, flavones, isoflavones, and pterocarpans. Flavanones, flavonols, and pterocarpans were predominant in roots, whereas flavones were predominant in the shoots. Isoflavones were identified in both the roots and shoot tissue of the legumes (Figure 3).

**Figure 4:**
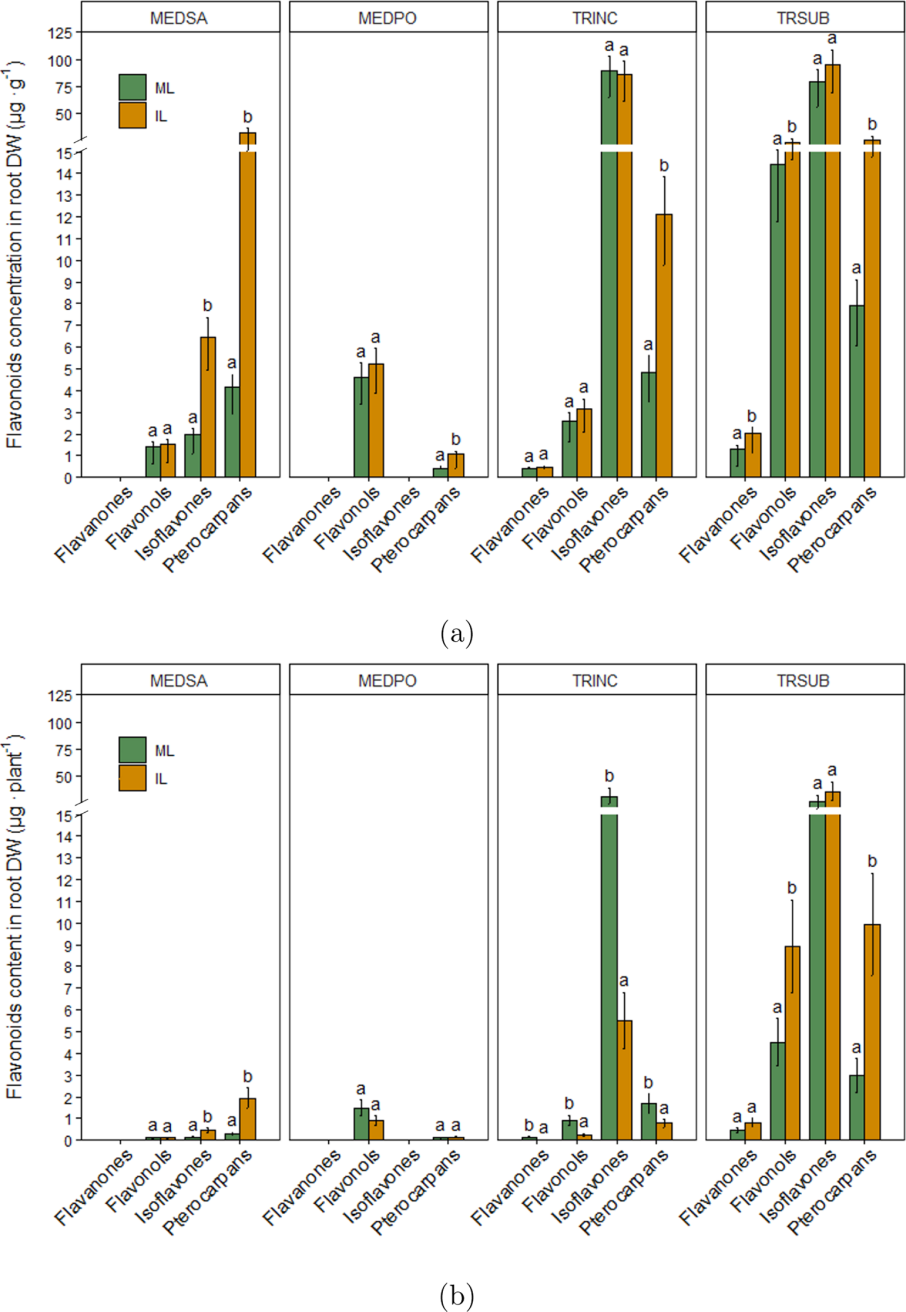
Flavonoid groups in legume root. (a) Concentration μg per g dry weight of root (b): content per plant. ML: monocropped legume, IL: intercropped legume. MEDSA: alfalfa, MEDPO: black medic, TRINC: crimson clover, and TRSUB: subterranean clover. Different letters (a,b) for each flavonoid group indicate significant differences at the 0.05 level (Tukey’s post-hoc test). Error bars represent S.E.

Co-cultivation significantly increased the concentration of isoflavones in alfalfa roots and the concentration of flavanones and flavonols in subterranean clover roots (Figure 4a). Total pterocarpan content significantly increased in intercropped alfalfa, black medic, crimson clover, and subterranean clover by about 4-, 3-, 3-, and 2-folds, respectively (Figure 4a). Despite the reduction observed in legume biomass during the co-cultivation with wheat, the total amount of pterocarpans per plant was still significantly higher in intercropped alfalfa and subterranean clover (about 7- and 4-folds, respectively) in comparison with the plants grown alone (Figure 4b).

**Figure 5:**
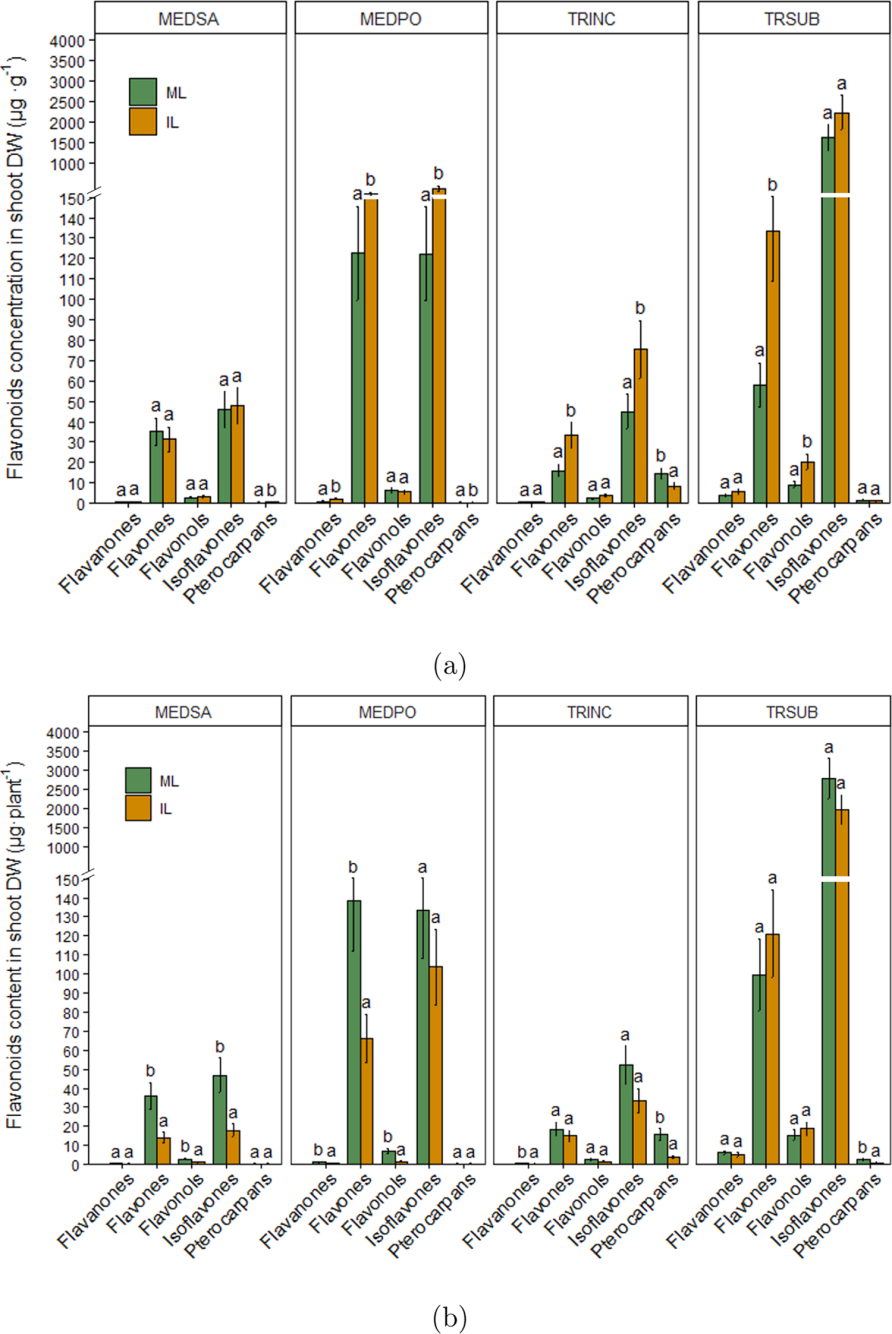
Flavonoid groups in legume shoot. (a) Concentration μg per g dry weight of shoot (b): content per plant. ML: monocropped legume, IL: intercropped legume. MEDSA: alfalfa, MEDPO: black medic, TRINC: crimson clover, and TRSUB: subterranean clover. Different letters (a,b) for each flavonoid group indicate significant differences at the 0.05 level (Tukey’s post-hoc test). Error bars represent S.E.

Co-cultivation also significantly increased the concentration of flavones in shoots of black medic, crimson clover, and subterranean clover compared to the corresponding legume species grown alone (Figure 5a). Isoflavones significantly increased in black medic and crimson clover, whereas an increase in the content of flavonols was observed in subterranean clover (Figure 5a). The flavonoid content of shoots per plant was not affected by wheat except for flavones in alfalfa and black medic and isoflavones in alfalfa, in which their concentration significantly decreased in co-cultivated plants (Figure 5b).

Fifteen flavonoid compounds were quantified in the root, shoot, and root exudates of the legumes. In this study, the detected flavonoids included kaempferol, astragalin, nicotiflorin, hyperoside, rutin, quercetin-rha-xyl-gal, apigenin, naringenin, daidzein, daidzin, formononetin, genistein, genistin, biochanin-A, and medicarpin (Table 2). Among these compounds, hyperoside, rutin, apigenin, formononetin, and naringenin were present exclusively in shoots, whereas the other compounds were identified in both root and shoot tissue.

**Table 2:** Common and Systematic Names of Compounds Quantified in Legumes in the Present Study and Their Frequently Reported Functional Role in the Rhizosphere

common name	systematic name	chemical group	functional role
Kaempferol	3,4,5,7-tetrahydroxyflavone	flavonols	Flavonols
Astragalin	kaempferol-3-*O*-glucoside		Nod regulator^18^
Nicotiflorin	kaempferol-3-*O*-rutinoside		Nematode repellent^19^
Hyperoside	quercetin-3-*O*-d-galactoside		Allelopathy^20^
Rutin	quercetin-3-*O*-rutinoside		Chelating agents^21,22^
Quercetin-Rha-Xyl-Gal	quercetin-3-*O*–d-rhamnosyl-(1 → 6)-d-xylosyl-(1 → 2)]--d-galactoside		
Apigenin	4,5,7-trihydroxyflavone	flavones	Nod regulator^23^
Naringenin	4,5,7-trihydroxyflavanone	flavanones	Nod regulator^24^
Daidzein	4,7-dihydroxyisoflavone	isoflavones	
Daidzin	daidzein-7-*O*-glucoside		Nod regulator^18,25,26^
Formononetin	7-hydroxy-4-methoxyisoflavone		Chelating agents^21^
Genistein	4,5,7-trihydroxyisoflavone		Allelopathy^6,27^
Genistin	genistein-7-*O*-d-glucoside		Nematode repellent^19^
Biochanin-A	5,7-dihydroxy-4-methoxyisoflavone		
Medicarpin	3-hydroxy-9-methoxypterocarpan	pterocarpan	Antimicrobial phytoalexin^28^
			Nod regulator^29^
			Allelopathy^6^

**Figure 6:**
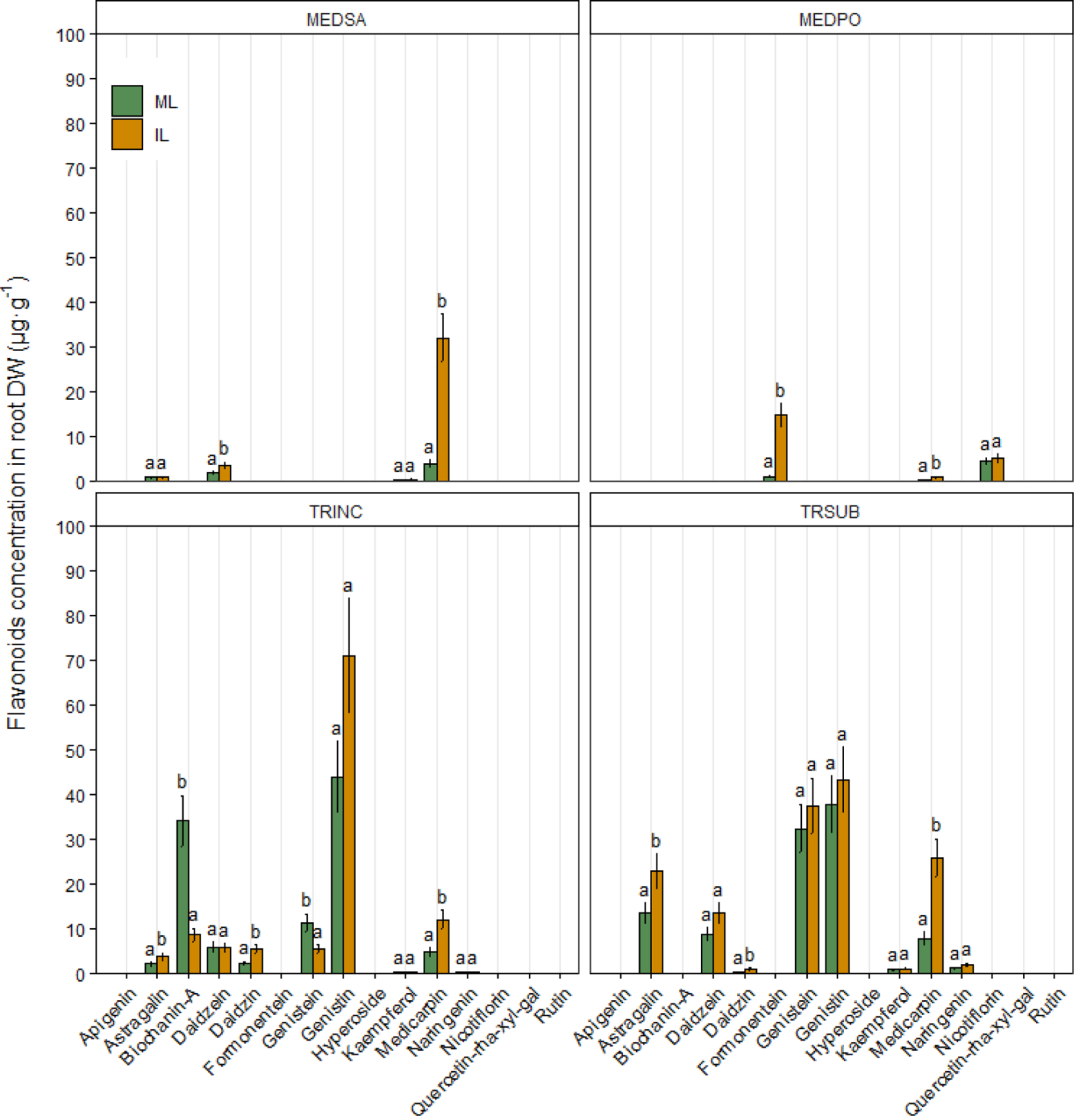
Effects of wheat-legume co-cultivation on the concentration of flavonoid compounds detected in root biomass (mean ± SE, μg·g^–1^ DW) of alfalfa (MEDSA), black medic (MEDPO), crimson clover (TRINC), and subterranean clover (TRSUB). ML: monocropped legume, IL: intercropped legume. Different letters (a,b) for each chemical compound indicate significant differences at the 0.05 level (Tukey’s post-hoc test). Error bars represent S.E.

The content of flavonoids varied among the legume species studied in this study. The predominant flavonoids in the root of alfalfa were astragalin, daidzein, kaempferol, and medicarpin. Among these, the concentration of daidzein and medicarpin significantly increased in response to co-cultivation with wheat (Figure 6).

Formononetin, medicarpin, and nicotiflorin were present in the root of black medic, and the co-cultivation with wheat significantly increased the root concentration of formononetin and medicarpin (Figure 6).

Astragalin, biochanin-A, daidzein, daidzin, genistein, genistein, kaempferol, medicarpin, and naringenin were detected in the roots of crimson clover. The concentrations of astragalin, daidzin, and medicarpin significantly increased in response to co-cultivation with wheat (Figure 6). In contrast, the concentrations of biochanin-A and genistein were significantly lower in intercropped plants (Figure 6).

Astragalin, daidzein, daidzin, genistein, genistin, kaempferol, medicarpin, and naringenin were detected in the roots of subterranean clover. Among them, the concentrations of astragalin, daidzin, and medicarpin significantly increased in intercropped plants (Figure 6).

**Figure 7:**
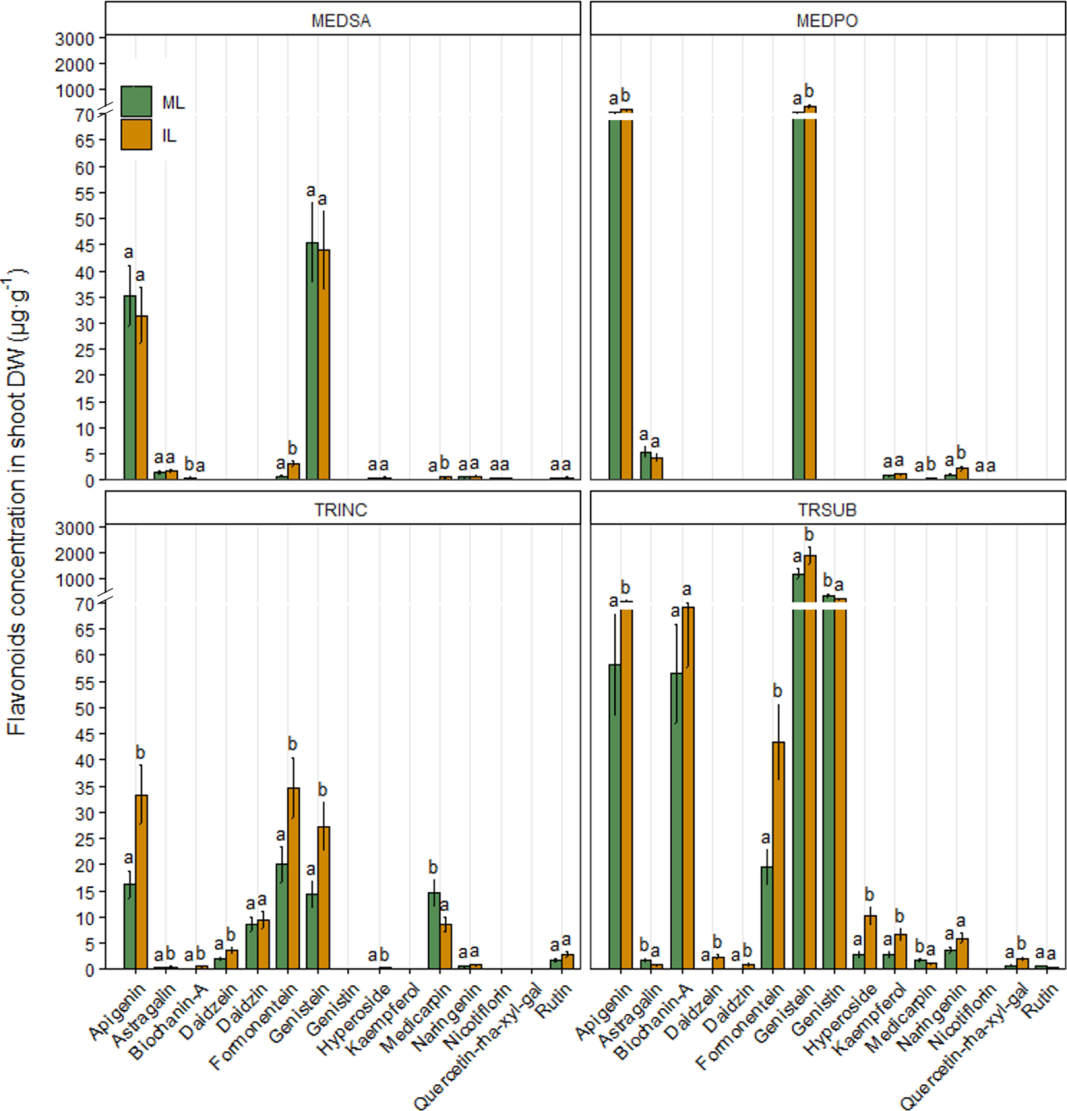
Effects of wheat-legume co-cultivation on the concentration of flavonoid compounds detected in shoot biomass (mean ± SE, μg·g^–1^ DW) of alfalfa (MEDSA), black medic (MEDPO), crimson clover (TRINC), and subterranean clover (TRSUB). ML: monocropped legume, IL: intercropped legume. Different letters (a,b) for each chemical compound indicate significant differences at the 0.05 level (Tukey’s post-hoc test). Error bars represent S.E.

The diversity of flavonoid compounds was higher in shoots than in roots. In alfalfa shoots, the concentrations of formononetin and medicarpin significantly increased in response to the co-cultivation with wheat compared with that found in plants grown in monoculture (Figure 7).

Flavonoids present in black medic included apigenin, astragalin, formononetin, genistein, kaempferol, medicarpin, naringenin, and nicotiflorin. In particular, the concentration of apigenin, genistein, medicarpin, and naringenin significantly increased in response to co-cultivation with wheat (Figure 7).

In the shoot of crimson clover, a significant increase in the concentration of apigenin, astragalin, biochanin-A, daidzein, formononetin, genistein, and hyperoside in response to co-cultivation with wheat was observed. In contrast, the concentration of medicarpin was negatively affected (Figure 7).

Apigenin, astragalin, biochanin-A, daidzein, daidzin, formononetein, genistein, genistin, hyperoside, kaempferol, medicarpin, naringenin, quercetin-rha-xyl-gal, and rutin were identified in the shoot of subterranean clover. The concentrations of apigenin, daidzein, daidzin, formononetin, genistein, hyperoside, kaempferol, and quercetin-rha-xyl-gal significantly increased in response to co-cultivation with wheat, whereas the concentrations of astragalin, genistin, and medicarpin were significantly lower in co-cultivated subterranean clover (Figure 7).

## Discussion

While flavonoids have been thoroughly investigated in most of the common forage legumes used in agriculture,^14^ it is still unclear how their content in plants alters in response to the co-cultivation with other crop species.^30^ This study aimed to investigate how the flavonoid profiles changed in root and shoot tissue and root exudates of four target legume species (alfalfa, black medic, crimson clover, and subterranean clover) in response to co-cultivation with durum wheat and therefore provide possible mechanistic explanations of the multiple beneficial effects reported at the cropping system level of legume-cereal intercropping.

Results from this study revealed that interspecific competition with wheat negatively affected legume growth and led to a significant reduction in shoot and root biomass compared with the same legume species grown in monoculture. In contrast, the concentration of flavonoids significantly increased both in legume biomass and root exudates, suggesting that in an intercropping system, plants can modulate the growth and production of secondary metabolites in response to interspecific competition, resulting in morphological and chemical plasticity.^3,30,31^

According to the results of this experiment, it was therefore suggested that wheat was the dominant species in the wheat/legume co-cultivation system due to the faster growth and higher competitiveness for light, space, and nutrients. Likewise, legumes sensed and responded to neighboring wheat by changing the chemical profile of their roots and their root exudate.^32−34^ Other studies report an increase of secondary metabolite production by legumes such as alfalfa, faba bean, or hairy vetch in response to co-cultivation with cereals such as wheat, rye, or triticale.^3,4,35,36^

Most of the changes in flavonoid concentrations detected in this study involved metabolites known to play an important role in the nodulation process and the regulation of interplant interaction. We speculate that increased competition for resources caused by the presence of wheat stimulated the biosynthesis and root exudation of flavonoids that promote the nodulation of legumes (nod-inducer compounds) such as daidzein,^25^ genistein,^18^ and medicarpin.^29^ These results are consistent with the findings from previous experiments conducted both in soil and hydroponic systems, showing that co-cultivation between cereal and legumes significantly increased the concentration of flavonoids in legume root exudate and promoted nodulation in legumes.^24,36^ Li et al., 2009, reported that soil nitrogen deficiency could stimulate the biosynthesis of nod-inducer flavonoids and regulate their release into the root zone through the mechanism known as negative feedback regulation of nitrogen.^35^ When legumes are used in intercropping systems with a cereal, rhizosphere N availability decreases during the first growing stages because of the stronger competition of cereals for nutrients. Nitrogen deficiency leads to greater production of flavonoids to stimulate nodulation and, thus, compensate for the soil nitrogen deficiency via fixation of atmospheric N.^4^ In this way, cereals can also benefit from the interspecific interaction with legumes through uptake of part of the N fixed by the legumes.

Many studies revealed that interactions between plant species depend mainly on resource competition,^4,24^ but there is increasing evidence showing involvement of allelochemicals.^37,38^ Also, when optimal nutritional conditions occur, plants sense and respond to neighboring plants’ roots by changing the chemical profile of their root and root exudates.^24,36,37^ Many studies have reported that flavonoids are important chemical mediators involved in allelopathic interactions in the soil rhizosphere^6^ and that interspecific plant co-existence may alter their production and release into the environment.^30,37,38^ Results from the glass bead experiment seemed to support this hypothesis. In the glass bead experiment, plants were regularly irrigated with a nutrient solution, and competition for nutrients between wheat and legumes can thus be considered very low. Although nutrients were not limiting plant growth in the glass bead experiment, a significant increase of flavonoids in the legume root exudates was detected, suggesting that the presence of wheat, perceived by the legumes as a competitor, could have triggered and stimulated the release of functional compounds in root exudates such as medicarpin and genistein.

The results of this study showed that the co-cultivation of legumes with wheat increased the concentration of flavonoids with growth inhibitory activity such as formononetin^6^ and medicarpin^6^ with potential beneficial implications for pest management, such as natural weed and arthropod pest control.^6,20^ This aspect can be particularly relevant for a living mulch system where legumes are introduced with the specific aim to contribute to weed control. However, in a living mulch system, the allelopathic effects of legumes are expected only to be directed against weeds and without negative impacts on the main crop.^39,40^ For a selective inhibitory effect on weeds, the use of crops with big seeds such as wheat, corn, sunflower, or a delayed undersowing of legumes (relay intercropping) can be recommended because the efficiency of allelochemical compounds depends on seed size and the phenological stage of the target plant.^41^

The characterization of legumes based on the flavonoid content can be an additional parameter to consider for the evaluation of suitable legumes for intercropping systems. Indeed, flavonoid profiles vary significantly between legume species and cultivars as well as their reaction to the co-cultivation with non-legume species.^14^ The results of this study showed that in legume root exudates, a significant increase in nod-inducer flavonoids such as genistein and medicarpin occurred in alfalfa, crimson clover, and subterranean clover, whereas no significant changes were found for black medic. The concentration of flavonoids also increased in legume biomass, and subterranean clover had the highest concentration of flavonoids both in the root and shoot compared with the other legumes in test. The concentration of daidzein and medicarpin significantly increased in alfalfa root biomass in response to co-cultivation. Moreover, the significant increase of genistein, combined with the reduction of nod-inhibiting flavonoids such as biochanin-A^26^ observed in crimson clover, may likely promote nodulation in this species when they are grown in an intercropping system.

In the legumes used in this experiment, the concentration of both medicarpin and formononetin significantly increased in response to co-cultivation with wheat in plant tissue and root exudates. In the root exudates, medicarpin was detected in all the legume species used in this study, whereas formononetin was detected only in alfalfa, crimson clover, and subterranean clover. In alfalfa, black medic, crimson clover, and subterranean clover, medicarpin was prevalently detected in legume root biomass, whereas formononetin was prevalently detected in shoot biomass, suggesting the close relationship between these chemical compounds. Formononetin has been reported to be a precursor of medicarpin.^42^ In particular, medicarpin was one of the most responsive flavonoid compounds to co-cultivation with wheat. It is known that medicarpin is an important nod-inducer compound, as well as powerful antimicrobial compound.^28^ Many of the nod-inducing flavonoids also act as antimicrobials that, along with other symbiosis nod-inducing flavonoids, may have a role in rhizosphere selection of compatible rhizobia and may be important determinants of host range in the field.^43^ In particular, medicarpin is a nod-inducer compound for *Rhizobium meliloti*, the specific nitrogen-fixing bacteria for alfalfa.^29^ Therefore, the increase in the concentration of medicarpin in root and root exudates of alfalfa observed in this study can primarily be assigned to the species-specific role of medicarpin on the alfalfa nodulation process.

Future studies should elucidate the bioactivity of flavonoid compounds in cereal-legume co-cultivation systems and their specific role in the nodulation process and inter-specific plant interactions to determine their potential and actual effects on weeds.
